# Analysis of HAX-1 gene expression in esophageal squamous cell carcinoma

**DOI:** 10.1186/1746-1596-8-47

**Published:** 2013-03-25

**Authors:** Min Li, Yue Tang, Wenqiao Zang, Xiaoyan Xuan, Na Wang, Yunyun Ma, Yuanyuan Wang, Ziming Dong, Guoqiang Zhao

**Affiliations:** 1 Department of Microbiology and Immunology, College of Basic Medical Sciences, Zhengzhou University, Zhengzhou, 450001, People’s Republic of China; 2 Department of Immunology and Microbiology, Henan Medical College for Stuff and Workers, Zhengzhou, 451191, People’s Republic of China; 3 Department of Pathophysiology, College of Basic Medical Sciences, Zhengzhou University, Zhengzhou, 450001, People’s Republic of China

**Keywords:** Esophageal squamous cell carcinoma, Gene expression, HAX-1, Prognosis

## Abstract

**Objective:**

To explore the expression of HAX-1 mRNA and protein in esophageal squamous cell carcinoma (ESCC) and its relation with the prognosis of patients with ESCC.

**Methods:**

The expression of HAX-1 mRNA and protein were detected with quantitative real-time RT-PCR and immunohistochemical method in 112 ESCC samples and 112 corresponding non-neoplastic samples. Survival curves were made with follow-up data. The relations of the prognosis with clinical and pathological characteristics were analyzed.

**Results:**

The expression level of HAX-1 mRNA and the strong positive rate of HAX-1 protein were significantly higher in ESCC samples (0.527 ± 0.060 and 45.54%) than that in non-neoplastic samples (0.121 ± 0.017 and 0.00%), and in ESCC samples with lymph node metastasis (0.554 ± 0.054 and 71.11%) than that in ESCC samples without lymph node metastasis (0.509 ± 0.058 and 28.36%) (all *P* < 0.01). HAX-1 mRNA expression level was a risk factor of lymph node metastasis in patients with ESCC (*P* = 0.000). There were significant differences in survival curves between lymph node metastatic group and non-metastatic group (*P* = 0.000), and among groups of HAX-1 protein expression +, ++and +++(,*P* = 0.000); but no statistical significance between male patients and female patients (*P* = 0.119), and between ≥60 years old patients and <60 years old patients (*P* = 0.705). The level of HAX-1 mRNA (*P* = 0.000) and protein (*P* = 0.005) were risk factors of survival, but lymph node metastasis (*P* = 0.477) was not.

**Conclusion:**

There is HAX-1 over-expression in ESCC tissue and HAX-1 mRNA level is a risk factor of lymph node metastasis. The level of HAX-1 mRNA and protein were risk factors of survival in patients with ESCC. HAX-1 may be a novel therapeutic target for ESCC treatment.

**Virtual slides:**

The virtual slide(s) for this article can be found here: http://www.diagnosticpathology.diagnomx.eu/vs/5130393079296037

## Introduction

HS1-associated protein X-1 (HAX-1) which can interact with hematopoietic cell specific Lyn substrate 1 (HS1), a Src kinase substrate involved in the maturation of T cells, was originally identified by Suzuki et al. [1] with yeast two-hybrid system in 1997. The human HAX-1 gene is located on chromosome 1 (1q21.3) [2]. HAX-1 is ubiquitously expressed in murine and human tissues, with the highest expression levels found in metabolically active tissues, particularly the skeletal and heart muscles, followed by substantial but evidently lower levels in brain and pancreas, whereas the kidney, liver, lung, and placenta contained the least amounts. In addition to the predominantly mitochondrial distribution, HAX-1 can also be found at nuclear membrane and endoplasmic reticulum [1].

HAX-1, as a kind of multifunctional protein, is involved in a variety of important physiological and pathological processes including anti-apoptosis, cell migration and endocytosis, and combination with mRNA 3’untranslated region (3^′^UTR) [3-11]. HAX-1 is related to genesis, invasion and metastasis of many tumors [12,13], and is over-expressed in a variety of tumors [12,14,15] such as oral squamous cell carcinoma [16], lung cancer [17], lymphoma, melanoma [12], leukemia, myeloma, breast cancer and hepatoma [18].

There is a high incidence of esophageal cancer in Henan Province. To explore the role of HAX-1 in esophageal squamous cell carcinoma (ESCC) and to provide a basis for finding new anti-esophageal cancer drugs, we detected the expression of HAX-1 mRNA and protein with real-time RT-PCR and immunohistochemical method, and made the survival analysis in 112 patients with ESCC.

## Materials and methods

### Patients and specimens

Between 2003 and 2005, 112 patients with ESCC were enrolled in this study from Tumor Hospital of Linzhou City, Linzhou People’s Hospital and the First Affiliated Hospital of Zhengzhou University. All the patients recruited to this study had not received any chemoradiotherapy, radiotherapy and immunotherapy directed against ESCC prior to oesophagectomy. Of the 112 patients, 62 were female and 50 were male, with a mean age of (59.58 ± 8.57) years (range 44–77). And there were 45 cases with lymph node metastasis, 67 cases without lymph node metastasis. ESCC and adjacent non-neoplastic samples (taken away from the tumor edge over 5 cm) were collected from each patient. Samples were quickly stored in liquid nitrogen for future use. This study was approved by the ethics committee of Zhengzhou University and informed consent was obtained from each patient.

### Primers

HAX-1 and β-actin primers were designed according to HAX-1 mRNA (NM_006118) and β-actin mRNA (NM_001101) with Oligo 6.0 software. The sequences were as follows: HAX-1 sense 5^′^-GACACTTCGGGACTCAATGCT-3^′^, HAX-1 antisense 5^′^-TAGGACTGCTATCTGCTTCGT-3^′^, β-actin sense 5^′^-CGGGACCTFACTGACTACCTC-3^′^, β-actin antisense 5^′^-CAAGAAAGGGTGTAACGCAAC- 3^′^. The length of fragments amplified by PCR with HAX-1 primers and β-actin primers were 380 bp and 618 bp, respectively. All the primers were synthesized by Shanghai Biosune Biotechnology Company.

### Real-time fluorescent quantitative RT-PCR

RNAs were extracted from 112 ESCC samples and 112 non-neoplastic samples with RNA extraction kit (Qiagen) and then cDNAs were generated by AMV (Promega). Real-time PCR was performed using SYBR® Green Real time PCR Kit (TaKaRa). PCR cycling condition was set as follows: an initial denaturing step at 95°C for 3 min and 35 cycles at 95°C for 20 s, 60°C for 60 s. CT value in each tube was recorded to calculate gene copy number. The housekeep gene, β-actin, was used as an internal control and normalization analysis. The comparative expression levels were determined as a ratio between HAX-1 and β-actin to correct for variation in the amounts of mRNA.

### Immunohistochemistry

112 ESCC samples and 112 non-neoplastic samples were fixed with 10% neutral buffered formalin, embedded in paraffin, and then sectioned. UltraSensitive^TM^ SP kit (Maxim-Bio, China) was employed for immunochemical staining according to the manufacturer’s instructions. Rabbit anti-HAX-1 antibodies (Santa Cruz) were applied at 1:50 dilution. The sections were stained with streptavidin peroxidase (SP) kit(Maixin Biotechnology Company, China), visualized with DAB coloration kit (Boaosen Company, China), followed by counterstaining of campeachy, dehydration, transparency and mounting. All slides were assessed by two observers independently and then in conference in a blinded manner without any prior knowledge of the clinicopathological parameters. Negative controls of immunohistochemical reactions were performed by omitting the primary antibody. Replacement of primary antibody by PBS was used as blank control.

Immunostaining of each section was semiquantitatively scored for intensity (0,absent;1,weak; 2, moderate; 3, strong) and extent of staining (percentage of the positive tumor cells: 0, ≤5%; 1, 6-25%; 2, 26-50%; 3, 51-75%; 4, >75%). The scoring results of intensity and extent were multiplied to give a composite score ranging from 1 to 12 for each section: 0,-(negtive); 1–4,+(weak positive); 5–8,++(moderate positive); 9–12,+++(strong positive).

### Follow-up and survival analysis

Patients were followed up. The overall survival period was defined as the duration from the postoperative time point to death time point. The follow-up deadline was March 6, 2010. The median follow-up time was 53 months (range 8–84). Based on the follow-up data, the survival curves were made. The relations of HAX-1 expression with age, sex and lymph node metastasis were analyzed.

### Statistical analysis

Statistical analysis was performed using SPSS13.0 software. Data were expressed as ($\bar{x}\;\pm \;\mathrm{SD}$). Student’s *t* test was used in the comparison of mean between two samples. Fourfold table Chi square test was used in the comparison of ratios between two samples. Logistic analysis was used in the correlation of lymph node metastasis with HAX-1 mRNA expression. The follow-up data was analyzed by the Kaplan-Meier method and log-rank test. Cox proportional hazards model were used in multivariate prognostic analysis. *P* values less than 0.05 were considered statistically significant.

## Results

### HAX-1 mRNA is over-expressed in ESCC samples and is a risk factor of lymph node metastasis

The relative expression level of HAX-1 mRNA was significantly higher in ESCC samples (112 samples, 0.527 ± 0.060) than that in non-neoplastic samples(112 corresponding samples, 0.121 ± 0.017) (*t* = -69.347, *P* = 0.000), and in ESCC samples with lymph node metastasis (45 samples, 0.554 ± 0.054) than that in ESCC samples without lymph node metastasis (67 samples, 0.509 ± 0.058) (*t* = 4.240, *P* = 0.000) (Table 1). Logistic regression analysis indicated that the relative expression level of HAX-1 mRNA was a risk factor of lymph node metastasis in the patients with ESCC (Wald *χ*^2^ = 12.743, *P* = 0.000).

**Table 1:** **Expressions of HAX-1 mRNA in ESCC samples (**$\bar{x}\pm S$**)**

**Groups**	**n**	**HAX-1 mRNA**	***P***
Peri-cancerous samples	112	0.121 ± 0.017	*P* = 0.000
ESCC samples	112	0.527 ± 0.060	
Age(yr)			
≥60	50	0.537 ± 0.063	*P* = 0.101
<60	62	0.519 ± 0.057	
Gender			
Male	50	0.535 ± 0.060	*P* = 0.231
Female	62	0.521 ± 0.060	
Lymph node metastasis			
Yes	45	0.554 ± 0.054	*P* = 0.000
No	67	0.509 ± 0.058	

### Strong positive rate of HAX-1 protein is high in ESCC samples and associated with lymph node metastasis

**Figure 1:**
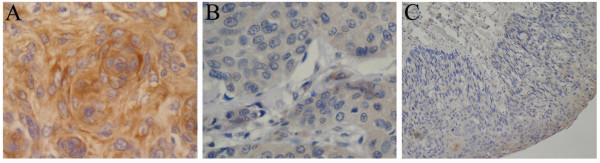
**All the ESCC samples and non-neoplastic samples were fixed with 10% neutral buffered formalin, embedded in paraffin, and then sectioned.** Then immunohistochemistry was carried out. Figure 1**A**: HAX-1 staining is strong positive in ESCC samples (400×); Figure 1**B**: HAX-1 staining is weakly positive in ESCC samples (400×); Figure 1**C**: HAX-1 staining is weakly positive in non-neoplastic samples (200×).

HAX-1 showed positive immuno-reactivity mainly in cytoplasms of the cells (Figure 1). The positive rate of HAX-1 protein expression was 100% in ESCC samples and corresponding non-neoplastic samples. The strong positive rate of HAX-1 protein expression was significantly higher in ESCC samples (45.54%, 51/112) than that in non-neoplastic samples (0.00%, 0/112), and in ESCC samples with lymph node metastasis (71.11%, 32/45) than that in ESCC samples without lymph node metastasis (28.36%, 19/67) (*P* < 0.05). There were no statistical differences between different sexes (male and female) and ages (≥60 years and < years) (Table 2).

**Table 2:** Expressions of HAX-1 protein in ESCC and non-neoplastic samples

**Groups**	**n**	**HAX-1 protein**				**Strong positive expression rate (%)**	***P***
		**-**	**+**	**++**	**+++**		
Peri-cancerous samples	112	0	61	51	0	0.00	*P* = 0.000
ESCC samples	112	0	9	52	51	45.54	
Age(yr)							
≥60	50	0	3	24	23	46.00	*P* = 0.929
<60	62	0	6	28	28	45.16	
Gender							
Male	50	0	3	25	22	44.00	*P* = 0.769
Female	62	0	6	27	29	46.77	
Lymph node metastasis							
Yes	45	0	0	13	32	71.11	*P* = 0.000
No	67	0	9	39	19	28.36	

### Expression level of HAX-1 mRNA and protein are risk factors of survival in patients with ESCC

**Figure 2:**
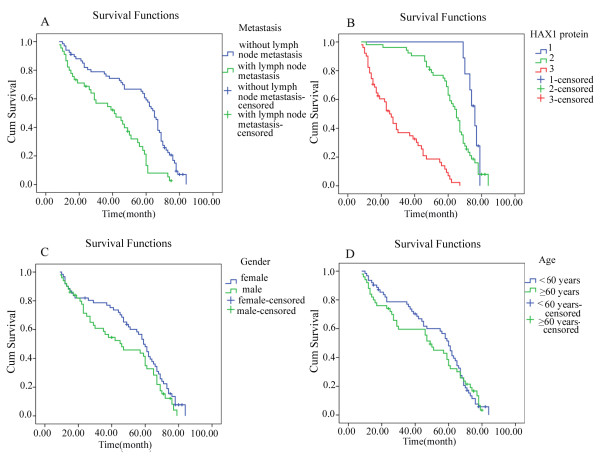
**The follow-up data was analyzed by the Kaplan-Meier method and log-rank test.** The survival curves were made. Figure 2**A**: Schematic representation shows survival curves of lymph node metastatic group and non-metastatic group in patients with ESCC. Figure 2**B**: Schematic representation shows survival curves of different protein level groups in patients with ESCC. Figure 2**C**: Schematic representation shows survival curves of different sexes in patients with ESCC. Figure 2**D**: Schematic representation shows survival curves of different ages in patients with ESCC. Log-rank test indicated that there were significant differences in survival curves between lymph node metastatic group and non-metastatic group (*χ*^2^ = 19.484, *P* = 0.000, Figure 2**A**), and among groups of HAX-1 protein expression +, ++ and +++ (*χ*^2^ = 80.729, *P* = 0.000, Figure 2**B**); but no significant differences between male patients and female patients (*χ*^2^ = 2.435, *P* = 0.119, Figure 2**C**), and between ≥60 years old patients and <60 years old patients (*χ*^2^ = 0.143, *P* = 0.705, Figure 2**D**).

Survival curves were drawn using SPSS13.0 software with Kaplan-Meier method. Log-rank test indicated that there were significant differences in survival curves between lymph node metastatic group and non-metastatic group (*χ*^2^ = 19.484, *P* = 0.000, Figure 2A), and among groups of HAX-1 protein expression +, ++ and +++(*χ*^2^ = 80.729,*P* = 0.000, Figure 2B); but no significant differences between male patients and female patients (*χ*^2^ = 2.435,*P* = 0.119, Figure 2C), and between ≥60 years old patients and <60 years old patients (*χ*^2^ = 0.143, *P* = 0.705, Figure 2D).

COX-univariate regression analysis indicated that lymph node metastasis (Wald *χ*^2^ = 17.967, *P* = 0.000), the expression level of HAX-1 mRNA (Wald *χ*^2^ = 91.507, *P* = 0.000) and the expression level of HAX-1 protein (Wald *χ*^2^ = 54.714, *P* = 0.000) were risk factors of survival in the patients with ESCC; but sex (Wald *χ*^2^ = 2.334, *P* = 0.127) and age (*P* = 0.711) were not risk factors of survival. Further COX-multivariate regression analysis indicated that the level of HAX-1 mRNA(Wald *χ*^2^ = 55.641,*P* = 0.000) and protein (Wald *χ*^2^ = 0.7.929, *P* = 0.005) were risk factors of survival, but lymph node metastasis (Wald *χ*^2^ = 0.506, *P* = 0.477) were not a risk factor of survival in the patients with ESCC.

## Discussion

HAX-1, a kind of multifunctional protein, is recently discovered [19]. It was first discovered in its interaction with HS-1 (Src kinase substrate), suggesting that HAX-1 is involved in B cell signal transduction. HAX-1 is associated with many cellular proteins and viral proteins, suggesting that HAX-1 is involved in multifunctional signaling pathways and cellular processes. The homology between HAX-1 and anti-apoptotic protein Bcl-2 suggests that HAX-1 is served as an apoptotic arrestin to participate in regulating apoptosis(namely programmed cell death); and also is involved in cell migration and endocytosis. It has been reported that HAX-1 silencing could induce melanoma cell apoptosis [20], suggesting that HAX-1 plays an important role in tumorigenesis and tumor metastasis [21]. Many molecules are involved in the metastasis of ESCC, such as ABCG2/V-ATPase [22].

Little research has been done on HAX-1 protein expression in ESCC samples. Previous studies of our team found that HAX-1 promotes the proliferation, chemo-resistance, invasion, and tumorigenicity of ESCC, and this is correlated with increased DNA polymerase β expression. HAX-1 may represent a potential target to overcome the resistance and metastasis of ESCC [23].

This study confirmed the presence of HAX-1 over-expression in ESCC samples for the first time. The expression level of HAX-1 mRNA and the strong positive rate of HAX-1 protein expression were significantly higher in ESCC samples (0.527 ± 0.060 and 45.54%) than that in non-neoplastic samples (0.121 ± 0.017 and 0.00%), and in ESCC samples with lymph node metastasis (0.5540 ± 0.054 and 71.11%) than that in ESCC samples without lymph node metastasis (0.509 ± 0.058 and 28.36%) (all *P* < 0.01). The expression level of HAX-1 mRNA was positively correlated with lymph node metastasis, and was a risk factor of lymph node metastasis in the patients with ESCC (Wald *χ*^2^ = 12.743, *P* = 0.000). There were significant differences in survival curves between lymph node metastatic group and non-metastatic group (*χ*^2^ = 19.484, *P* = 0.000), and among groups of HAX-1 protein expression +, ++ and +++(*χ*^2^ = 80.729, *P* = 0.000); but no significant differences between male patients and female patients (*χ*^2^ = 2.435,* P* = 0.119), and between ≥60 years old patients and <60 years old patients (*χ*^2^ = 0.143, *P* = 0.705). The level of HAX-1 mRNA (Wald *χ*^2^ = 55.641, *P* = 0.000) and protein (Wald *χ*^2^ = 0.7.929, *P* = 0.005) were risk factors of survival, but lymph node metastasis (Wald *χ*^2^ = 0.506, *P* = 0.477) was not a risk factor of survival in the patients with ESCC. These results suggest that there is HAX-1 over-expression in ESCC samples and the level of HAX-1 mRNA is a risk factor of lymph node metastasis and survival in the patients with ESCC. The results may provide a basis for exploring the role of HAX-1 in ESCC. The expression level of HAX-1 is expected to become an important index to assess ESCC invasion and metastasis, and prognosis of patients with ESCC. HAX-1 may be a novel therapeutic target for ESCC treatment.

## Conclusion

In conclusion, our data offer the convincing evidence that there is HAX-1 over-expression in ESCC tissue and the level of HAX-1 mRNA is a risk factor of lymph node metastasis. The level of HAX-1 mRNA and protein are risk factors of survival in patients with ESCC. HAX-1 may be a novel therapeutic target for ESCC treatment.

## Abbreviations

ESCC: Esophageal squamous cell carcinoma; HS1: Hematopoietic cell specific Lyn substrate 1; HAX-1: HS1-associated protein X-1

## Competing interests

The authors declare that they have no competing interests.

## Authors’ contributions

GQZ, ZMD and ML: conceived of the study, and participated in its design and coordination and helped to draft the manuscript. YYW and YT: collected the samples. ML, YYW, WQZ, and YYM: carried out part of experiments and wrote the manuscript. ML, NW and YT performed the statistical analysis. All authors read and approved the final manuscript.
